# Supplementary material for: CBL-Interacting Protein Kinases 18 (CIPK18) Gene Positively Regulates Drought Resistance in Potato
Source: Int J Mol Sci. 2023 Feb 10;24(4):3613. doi: 10.3390/ijms24043613 (PMC9964222; doi:10.3390/ijms24043613)
Supplement: Supplementary file 1 [file ijms-24-03613-s001.zip › ijms-2117000-supplementary.pdf]

Table S1. Primer information used in this study

Primer	Sequence	Usage
EGFP-StCIPK18-F	CGGGGGACGAGCTCGGTACCATGGAGAGTAAGGGGAGTAGTATAC	Subcellular localization
EGFP-StCIPK18-R	CCATGTCGACTCTAGATGCATTCTTCTGAGGTGAATTAGTG	
OE-StCIPK18-F	CGGGGGACGAGCTCGGTACCATGGAGAGTAAGGGGAGTAGT	Construction of overexpression vector
OE-StCIPK18-R	CCATGTCGACTCTAGATTATGCATTCTTCTGAGGTGAAT	
A	CTGCAAGGCGATTAAGTTGGGTAAC	Interference expression vector construction
B	GCGGATAACAATTTCACACAGGAAACAG	
I	gaTAGTGTCAAATGAGTACGCCTtctctcttttgtattcc	
II	gaAGGCGTACTCATTTGACACTAtcaaagagaatcaatga	
III	gaAGACGTACTCATTAGACACTTtcacaggtcgtgatatg	
IV	gaAAGTGTCTAATGAGTACGTCTtctacatatatattcct	
qPCR-CIPK18-F	GCAGCGATTGATGATGCAGG	qRT-PCR
qPCR-CIPK18-F	GATTGTTTTCGCCGGTTGCT	
EF1α-F	GATGGTCAGACCCGTGAACA	
EF1α-R	CCTTGGAGTACTTCGGGGTG	
HPT-F	GGTCGCGGAGGCTATGGATGC	HPT gene amplification
HPT-R	GCTTCTGCGGGCGATTTGTGT	
NPTII-F	GCTATGACTGGGCACAACAG	NPTII gene amplification
NPTII-R	ATACCGTAAAGCACGAGGAA	
BD-CIPK18-F	AGGAGGACCTGCATATGATGGAGAGTAAGGGGAGTAGT	Y2H
BD-CIPK18-R	GCCGCTGCAGGTCGACTTATGCATTCTTCTGAGGTGAAT	
AD-CBL1-F	CAGATTACGCTCATATGATGTTGTCGTGCTTAGGTTCTTACC	
AD-CBL1-R	TTCATCTGCAGCTCGAGCTCTCAATCCCAAATCAGGTCGTCC	
AD-CBL3-F	CAGATTACGCTCATATGATGGGCTGCTTTCACTCAAAAATC	
AD-CBL3-R	TTCATCTGCAGCTCGAGCTCCTAGACTTCCGAATCTTCCACCTC	
AD-CBL4-F	CAGATTACGCTCATATGATGGGCTGCTTTCACTCAAAAA	
AD-CBL4-R	TTCATCTGCAGCTCGAGCTCCTAAATTTCTGAATCATCAACCTCA	
AD-CBL6-F	CAGATTACGCTCATATGATGCTGCAGTTCTTAGGTTC	
AD-CBL6-R	TTCATCTGCAGCTCGAGCTCTTAGGTGTCCTCAACTCTTGAATG	
AD-CBL7-F	CAGATTACGCTCATATGATGGGCTGTTTTAGCTCTAAGGTGG	

AD-CBL7-R	TTCATCTGCAGCTCGAGCTCTCAAGTAGCTCCTTCATCAACTTC	
AD-CBL8-F	CAGATTACGCTCATATGATGGGGTGTGCTTTAAGGA	
AD-CBL8-R	TTCATCTGCAGCTCGAGCTCTCAGAAATCCTTGTAAATCTCATCA	
AD-CBL10-F	CAGATTACGCTCATATGATGGATTCCACGCGAAGTTCTC	
AD-CBL10-R	TTCATCTGCAGCTCGAGCTCTCACAACAAATGGGTTTTCTCCG	
AD-CBL11-F	CAGATTACGCTCATATGATGGGCTGCTTTAATTCTAAGGTGA	
AD-CBL11-R	TTCATCTGCAGCTCGAGCTCTTATGTAGCAACTTCATCAACTTCA	
AD-CBL12-F	CAGATTACGCTCATATGATGTCGTATTGCTTTGAGGGG	
AD-CBL12-R	TTCATCTGCAGCTCGAGCTCTCAAGTATCCGGAACTCTTGAGTG	
AD-CBL13-F	CAGATTACGCTCATATGATGTTGCAGTGCCTAGAGGGG	
AD-CBL13-R	TTCATCTGCAGCTCGAGCTCTCAGGTATCCTCAACTCTGGAATG	
BiFC-CIPK18-F	CACGGGGGACTCTAGAATGGAGAGTAAGGGGAGTAGT	BiFC
BiFC-CIPK18-R	TACATCCCGGGAGCGGTACCTTATGCATTCTTCTGAGGTGAAT	
BiFC-CBL4-F	CACGGGGGACTCTAGAATGGGCTGCTTTCACTCAAAAA	
BiFC-CBL4-R	TCCATCCCGGGAGCGGTACCAATTTCTGAATCATCAACCTCAGAG	

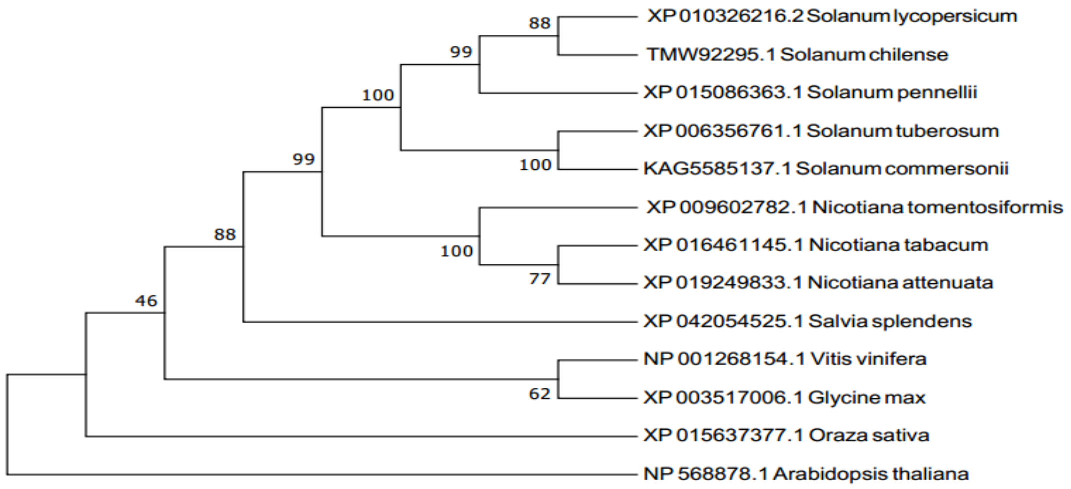

Figure S1. The phylogenetic tree analysis of StCIPK18 amino acid sequence with other species of homologous sequence

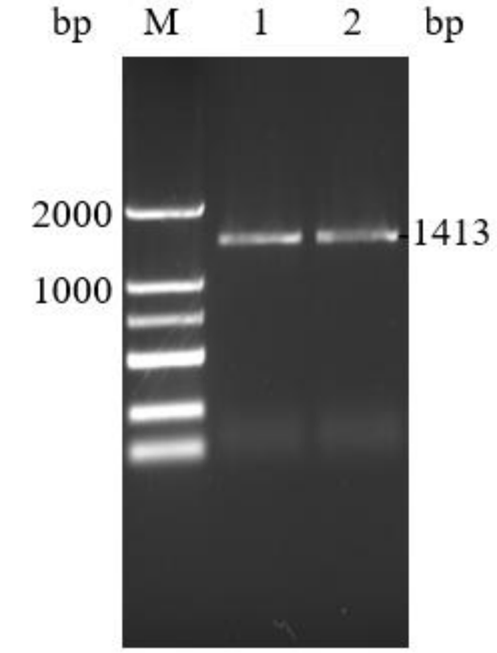

Figure S2. Result of PCR product of *StCIPK18* gene

M：DNA Maker DL2000; 1-2：*StCIPK18* Gene target segment
